# Comparative Efficiency of HIV-1-Infected T Cell Killing by NK Cells, Monocytes and Neutrophils

**DOI:** 10.1371/journal.pone.0074858

**Published:** 2013-09-10

**Authors:** Adjoa Smalls-Mantey, Mark Connors, Quentin J. Sattentau

**Affiliations:** 1 Sir William Dunn School of Pathology, University of Oxford, Oxford, United Kingdom; 2 HIV-Specific Immunity Section, Laboratory of Immunoregulation, National Institute of Allergy and Infectious Diseases, National Institutes of Health, Bethesda, Maryland, United States of America; University of Amsterdam, Netherlands

## Abstract

HIV-1 infected cells are eliminated in infected individuals by a variety of cellular mechanisms, the best characterized of which are cytotoxic T cell and NK cell-mediated killing. An additional antiviral mechanism is antibody-dependent cellular cytotoxicity. Here we use primary CD4^+^ T cells infected with the BaL clone of HIV-1 as target cells and autologous NK cells, monocytes, and neutrophils as effector cells, to quantify the cytotoxicity mediated by the different effectors. This was carried out in the presence or absence of HIV-1-specific antiserum to assess antibody-dependent cellular cytotoxicity. We show that at the same effector to target ratio, NK cells and monocytes mediate similar levels of both antibody-dependent and antibody-independent killing of HIV-1-infected T cells. Neutrophils mediated significant antibody-dependent killing of targets, but were less effective than monocytes or NK cells. These data have implications for acquisition and control of HIV-1 in natural infection and in the context of vaccination.

## Introduction

The primary target of HIV-1 infection *in vivo* are CD4^+^ T cells. Not only are these thought to be the first cells infected after sexual transmission, but they also account for up to 90% of the infected cell burden *in vivo* [1]. Control of established HIV-1 infection is mediated predominantly by CD8^+^ cytotoxic T cells [2], but there is also evidence that natural killer cells (NKs) play a role [3–5]. NKs can effect HIV-1 infected cell killing either directly, or via antibody-dependent cellular cytotoxicity (ADCC [6,7]). Although the role of ADCC in the control of HIV-1 infection is unclear, it does appear to contribute to the protective effect of antibodies (Abs) *in vivo* [8]. The role of monocytes (MCs) is less well established. Recent reports have demonstrated MC killing of an HIV-1-infected T cell line *in vitro* via an ADCC mechanism [9,10]. However, the target cells used in these assays were a recombinant antigen-coated immortalized cell line rather than infected autologous primary cells, and the relative efficiency of killing compared to NKs was not revealed. Polymorphonuclear neutrophils (PMNs) are innate immune cells with properties that include rapid infiltration into sites of inflammation, intrinsic phagocytic and other antibacterial activity, and expression of Fc receptors (FcRs) allowing mediation of Ab-mediated effector mechanisms [11]. Although PMNs are classically described as cells with activity against extracellular pathogens [12,13], they have antiviral activity and can reduce influenza virus infection in cell culture by ADCC-type effector mechanisms [14,15]. In addition, their function against HIV-1-infected cells has been proposed in the presence and absence of HIV-1-specific Abs [16,17].

The rapid recruitment of PMNs, and with somewhat delayed kinetics, MCs, to sites of inflammation mean that if these cells have antiviral activity then they may influence HIV-1 transmission and/or established viral replication [18]. Here we show that MCs have killing activity equivalent to NKs both in the absence and presence of HIV-1 specific Abs, and that PMNs can also kill HIV-1-infected T cells via an Ab-dependent mechanism. These results have important implications for the role of innate immune effector cells in the establishment and control of HIV-1 infection.

## Results

**Figure 1:**
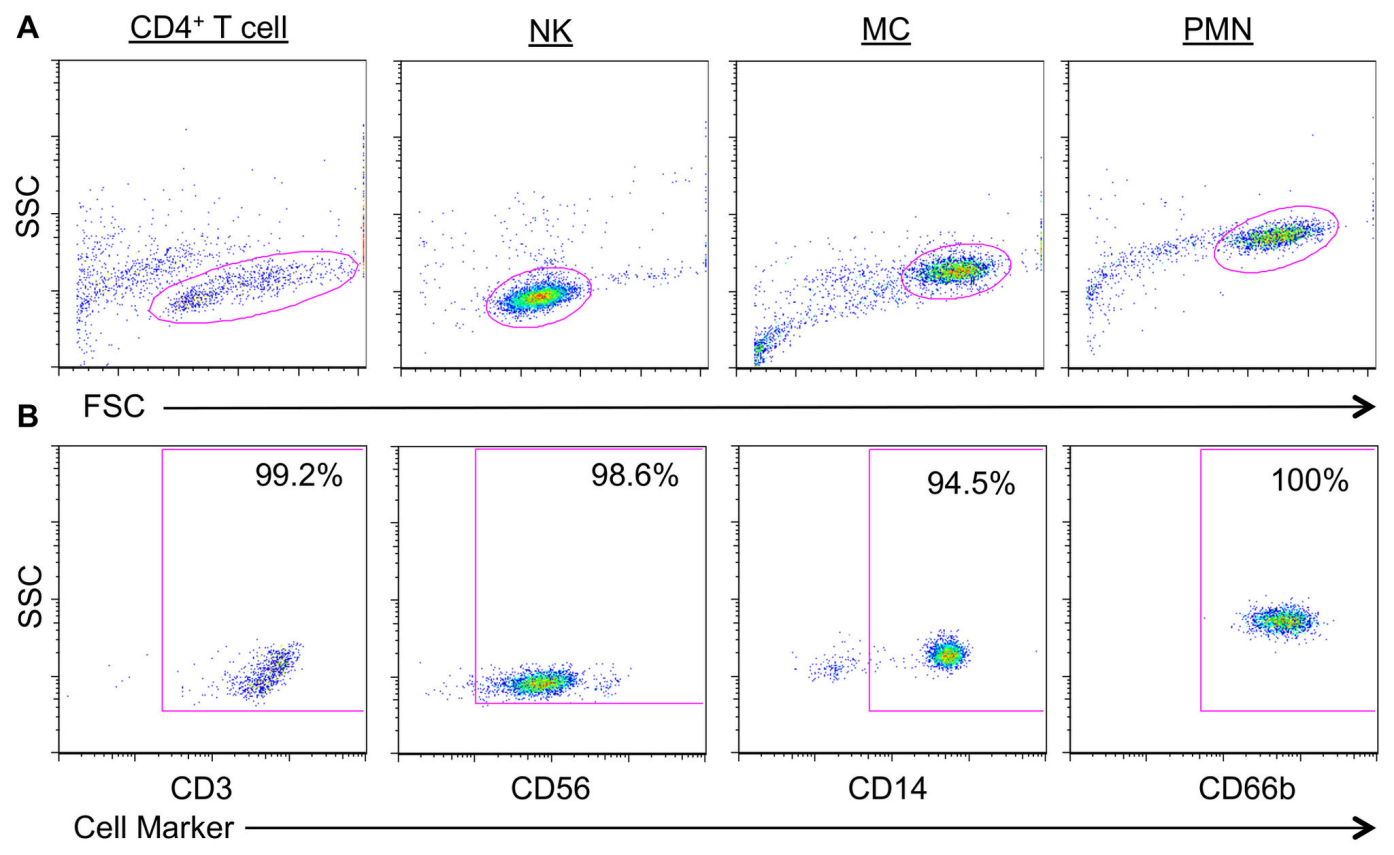
Purity of isolated target and effector cells. Isolated cells were stained with an appropriate single cell marker for identification. The percent positive populations (B) were assessed on live-gated cells (A): CD4^+^ T cell = CD3^hi^, NK = CD56^dim^, MC = CD14^hi^, PMN = CD66b^hi^.

Primary target and effector cells were isolated from PBMCs freshly obtained from seven normal healthy subjects and immediately prepared for the killing assay. Different cell subsets were isolated using magnetic bead-based negative selection, and purity assessed using flow cytometry and markers specific for the individual cell types: CD4^+^ T cell (CD3); NK (CD56); MC (CD14) and PMN (CD66b). Mean purity across seven experiments was as follows: CD3^+^CD4^+^ T cells = 99.4%, NKs = 98.7%, MCs = 92.5% and PMNs = 100% (Figure 1).

**Figure 2:**
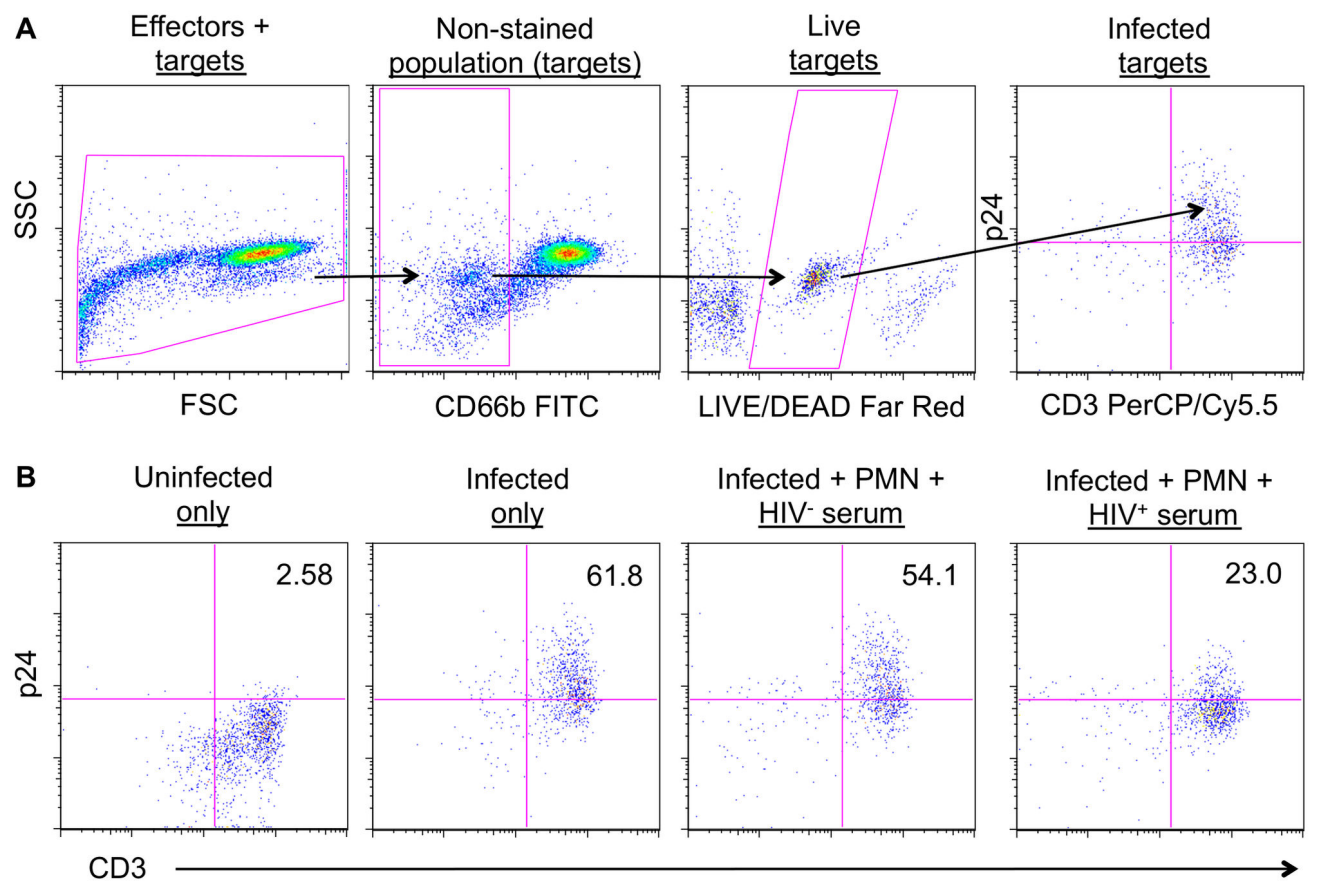
Gating strategy for ICE, Assay. (A) Effector cells were labeled with the phenotypic marker mAbs shown and targets labeled with LIVE/DEAD^®^ stain and mixed. Live CD4^+^ T cells (targets) were identified by gating on live cells negative for the effector label (NK, MC, or PMN-FITC). Cells were fixed and stained for p24 to determine the frequency of infected targets. (B) Elimination of HIV-1-infected cells. Representative data show % p24 positive cells for uninfected CD4^+^ T cells, infected CD4^+^ T cells, infected CD4^+^ T cells + PMN + HIV^-^ (control) serum, and infected CD4^+^ T cells + PMN + HIV^+^ (Bpool) serum. A reduction in the number of p24-stained cells is represented as % ICE and indicates cell death. In the example described above, % ICE_Infected CD4+PMN+Bpool_ = [(61.8-23.0)/61.8] × 100 = 62.8%.

The killing assay was based upon that previously described [19]. Briefly, CD4^+^ T cells were stimulated for 72 h and subsequently magnetofected™ with HIV-1_BaL_ for 48 h, yielding an average infection across seven experiments of 54.8%. Non-viable infected CD4^+^ T cell targets were excluded from analysis by selective labeling with a dead cell stain. Autologous effectors were isolated and combined at an effector:target ratio of 10:1 in the presence or absence of heat-inactivated human serum for 1 h at 37^o^C, fixed, permeabilized, and stained for intracellular viral Gag. Target cell killing (HIV-1 infected CD4^+^ T cell elimination - ICE), was evaluated by quantifying the loss of Gag^+^ CD3^+^ T cells from the live cell gate compared to Gag^+^ T cell loss in the absence of effector cells (Figure 2).

**Figure 3:**
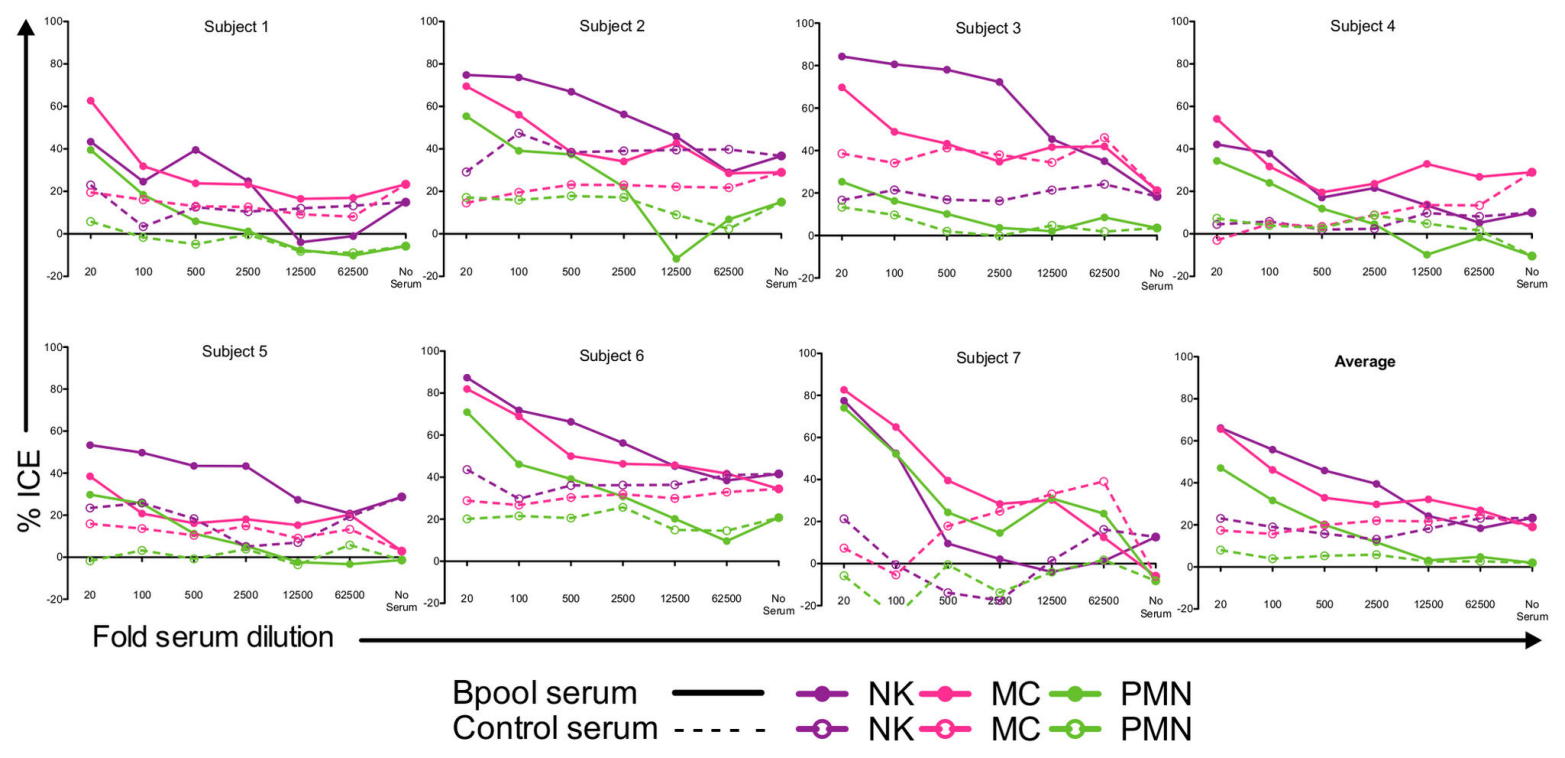
NKs, MCs, and PMNs mediate ICE. Percentage ICE mediated by NKs, MCs, and PMNs in seven subjects, at an effector:target ratio of 10:1 without or with Bpool (HIV^+^) or control (HIV^-^) serum at serial 5-fold dilutions starting at 1:20. Each datum point represents the mean of duplicate samples.

Killing was evaluated in seven subjects in the absence of human serum or using serum from an HIV-1^-^ donor (control serum) to assess non-ADCC-mediated killing, or in the presence of pooled HIV-1^+^ (Bpool) serum to assess ADCC. Serum was diluted serially 5-fold from 1:20 to 1:62,500 (Figure 3). There were inherent differences in the level of cytotoxicity mediated by subjects’ effector cells against HIV-1 infected CD4^+^ T cells (Figure 3, no serum) that was not significantly modulated by control serum. Killing efficiency varied substantially between donors. In some donors killing by all effector cell types was negligible in the absence of immune serum, whereas in others NK and MC killing varied from 20–40%. In general PMN killing in the absence of antiserum was absent or weak. Pooling the data from all donors revealed a clear increase in killing efficiency with increasing concentration of antiserum, with NKs and MCs yielding approximately equivalent killing of ~65% and PMNs mediating ~47% ICE at a serum dilution of 1/20.

**Figure 4:**
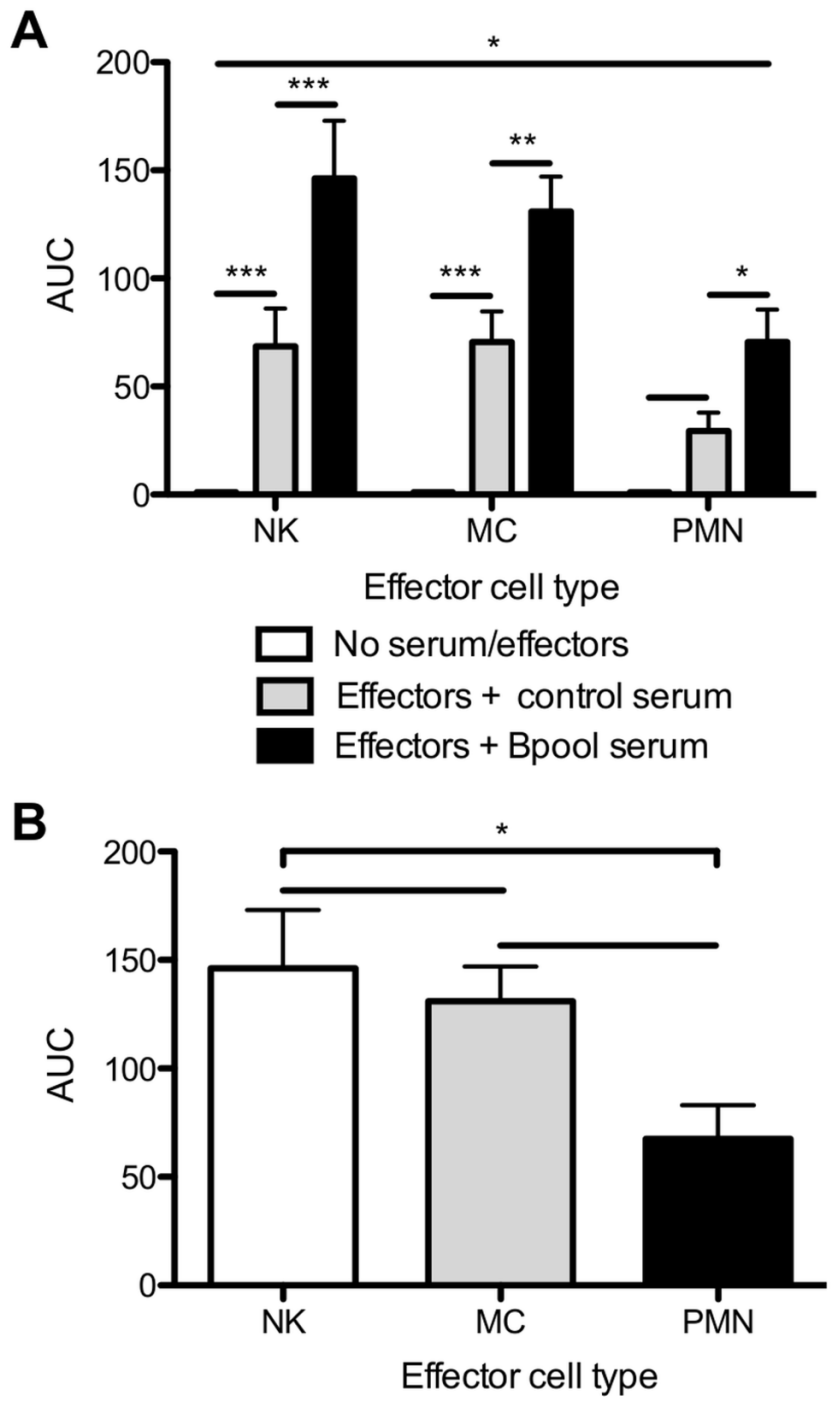
NKs, MCs, and PMNs differ in killing activity. Total area under the curve (AUC) of % ICE curves presented in Figure 3 for log_10_-transformed serum dilutions from 1:20 to 1:62,500 was calculated for all 7 subjects and means calculated. Comparisons of independent groups were made by two-way or one-way ANOVA (A and B, respectively) with Bonferroni post-test. (A) Comparison of AUC for infected cell elimination in the absence of serum or effectors (AUC set at 1), effector cells with control serum, and effector cells with Bpool serum. HIV-1-specific Abs enhance killing by NKs (*P* < 0.001), MCs (*P* < 0.01), PMNs (*P* < 0.05). (B) Comparison of AUC for effector cells with Bpool serum. NKs and MCs mediate similar levels of ADCC; PMNs mediate significantly less ADCC than NKs (*P* < 0.05).

These trends were confirmed by carrying out an area under the curve analysis of log-transformed x-axis curves of killing at all dilutions of antiserum or in the absence of antiserum in the combined data set. NKs and MCs alone had a highly significant intrinsic ability to kill infected cells (Figure 4A, no serum/effectors compared to effectors + control serum, *P* < 0.001) whereas PMNs showed a modest but insignificant trend to intrinsic killing (no serum/effectors compared to effectors + control serum *P* > 0.05). ADCC due to HIV-1-specific Abs titrated out at higher serum dilutions to yield values similar to killing of effectors with or without uninfected control serum (Figure 3). HIV-1 specific Abs in Bpool serum significantly enhanced killing by NKs (*P* < 0.001), MCs (*P* < 0.01), and PMNs (*P* < 0.05) (Figure 4A) when compared to control serum. NKs mediated statistically equivalent levels of killing to MCs, whereas PMNs mediated a significantly lower level of ADCC compared to NKs (*P* < 0.05) (Figure 4B).

## Discussion

Others have reported intrinsic killing and ADCC mediated by NKs, MCs, and PMNs in several different assays [9,16,17] but in this study we quantitatively compare the relative efficiency of these three effector cell types using a highly biologically relevant assay system. We show that NKs and MCs mediate similar levels of ADCC whereas PMNs mediate significantly less ADCC than NKs and MCs. These differences may be attributable to differing levels of FcγR expression, divergent mechanisms with different efficiencies of killing, or both. NKs only express activating FcγRIIIA (CD16a) [20] while MCs and PMNs in addition express both activating and inhibitory FcγRs, FcγRI (CD64) and FcγIIA/B (CD32a/b) [10,20,21]. Kramski et al. [10] observed more FcγR-IgG binding to PMNs and MCs than NKs. Although they observed greater IgG binding to PMNs, the mechanism was unclear since the relative expression of FcγRs and level of IgG binding to specific FcγRs was not shown. NK cytotoxicity mediated by perforin/granzyme and Fas/FasL has been well characterized [19,22]. However the mechanism by which PMNs and MC mediate ADCC is still undefined. Both MCs and PMNs use reactive oxygen species to kill bacteria and while this is generated in response to HIV-1 and during ADCC, it is not essential for killing [23–25]. MCs and PMNs also contain similar secretory granules such as CD63^+^ azurphilic granules [21], although arguably not perforin and granzyme [26], which may have a role in ADCC. Neutrophil extracellular traps (NETs) have recently been identified as a mechanism by which PMNs kill fungi, bacteria, influenza-infected cells, and most recently, HIV-1 [23]. It could be that PMNs exhibit less intrinsic killing and ADCC than NKs or MCs because they use NETs resulting from lysis of PMNs, which may be a less cell-specific and efficient mode of killing.

PMNs and MCs need no priming to mediate their effector functions, and are the first cell types recruited to sites of inflammation. PMNs in particular are recruited very rapidly and in high numbers, and so may make up for their reduced killing efficiency by increased effector: target ratios. The lamina propria of mucosal tissues are the first sites of viral replication after HIV-1 transmission, following this, the virus migrates to gut-associated lymphoid tissue (GALT) in which uncontrolled viral replication is associated with massive CD4^+^ T cell death and local inflammation [27]. Such an inflammatory response is likely to recruit PMNs and MCs [28]. Whether these cell types can modulate early HIV-1 infection by establishing an antiviral environment is an important question that remains to be addressed. In a prophylactic vaccine setting, in which HIV-1-specific Abs were raised, PMNs and MCs could participate in ADCC of HIV-1-infected cells during early infection, potentially increasing Ab effector functions. Thus understanding how innate effector cells kill HIV-1 infected cells will provide important insights into pathogenesis and may inform strategic vaccine designs that would allow us to harness their cytotoxic capacity in acute HIV-1 infection.

## Methods

### Cell isolation and characterization

Heparinized blood was collected from seven HIV^-^ donors and leukocytes separated using Ficoll density centrifugation. NKs and MCs were negatively isolated from PBMCs using EasySep^®^ Human NK and Monocyte Kits (StemCell, Vancouver, BC, Canada). Granulocytes were enriched for using dextran sedimentation and hypotonic lysis and PMNs isolated from granulocytes using the Neutrophil EasySep^®^ Enrichment Kit. CD4^+^ T cells were positively selected for using MACS CD4 MicroBeads (Miltenyi, Bergisch Gladbach, Germany). The purity of cells was assessed for each experiment by indirect immunofluorescence and flow cytometry using the following mAbs: CD3 (UCHT1, BioLegend, San Diego, CA, USA), CD56 (MEM-188, BioLegend), CD14 (M5E2, BD Biosciences, Franklin Lakes, NJ), CD66b (G10F5, BioLegend). The MC fraction did not stain for CD3, CD8, or CD66 but had a small percentage of CD56 labeled cells, which may represent non-classical CD56^+^ MCs [29].

### Killing assay

Killing assays were performed as previously described with slight modification [19]. In brief, CD4^+^ T cells were OKT3/anti-CD28/IL-2 stimulated for 72 h and infected with HIV-1_BaL_ using ViroMag beads (OZ Biosciences, Marseille, France) for 48 h, reaching an average infection across experiments of 54.8%. On the day of the assay infected CD4^+^ T cell targets were consecutively labeled with LIVE/DEAD^®^ Fixable Far Red (Invitrogen, Eugene, OR, USA) and anti-CD3 PerCP/Cy5.5 (BioLegend). Autologous effectors were isolated and labeled with the aforementioned Abs: MCs, anti-CD14 FITC; NKs, anti-CD56 FITC; PMNs, anti-CD66b FITC. Cells were combined at an effector:target ratio of 10:1 in the presence or absence of heat-inactivated serum for 1 h at 37^o^C, fixed, permeabilized, and stained for intracellular viral capsid p24 using KC57-RD1 (Beckman Coulter, Miami, FL, USA). HIV^+^ (Bpool) serum was pooled from 20 donors infected with clade B HIV-1. HIV^-^ (control) serum was obtained from a single uninfected donor. Samples were analyzed by flow cytometry using a BD FACS Calibur™. Data were analyzed using FlowJo software (TreeStar, San Carlos, CA, USA) and statistical analysis performed with Prism® (GraphPad Software, La Jolla, CA, USA). The readout for the killing assays was HIV-1 infected CD4^+^ T cell elimination (ICE). ICE was calculated as [(% p24 expression of CD3^+^ infected targets – % p24 expression of CD3^+^ infected targets mixed with effectors and sera)/% p24 expression of CD3^+^ infected targets] × 100. Experiments were performed in duplicate for all donors.

### Ethics Statement

HIV-infected subjects were recruited from the Clinical Research Center, National Institutes of Health (Bethesda, MD), and signed National Institute of Allergy and Infectious Diseases (NIAID) Investigational Review Board (IRB)-approved informed consent documents. Blood was collected from uninfected donors in accordance with the University of Oxford Occupational Health Service guidelines; written and verbal consent was obtained from all donors. This study was approved by the NIAID IRB.
